# Editorial: Perspective challenges for applied research in potato pathogens: From molecular biology to bioinformatics

**DOI:** 10.3389/fmicb.2023.1140107

**Published:** 2023-02-28

**Authors:** Rachid Lahlali, Grace Gachara, Göksel Özer, Hussain Touseef

**Affiliations:** ^1^Ecole Nationale d'Agriculture de Meknès, Meknès, Morocco; ^2^Department of Agriculture, Fertilization and Environmental Sciences, Mohammed VI Polytechnic University, Ben Guerir, Morocco; ^3^Bolu Abant Izzet Baysal University, Bolu, Türkiye; ^4^Matimate Agromart Private Ltd. (Sevama AgriClinic Laboratory), Bhavnagar, India

**Keywords:** disease surveillance, point-of-care diagnostics, isothermal amplification, high-throughput sequencing, biosensors, bio-imaging, bioinformatics, pathogenicity

The potato crop is prone to infection by an estimated 50 different categories of pests and diseases whose causal agents are either viruses, bacteria, nematodes, insects, or fungi (Hussain and Singh, 2016; Jansson and Raman, 2019; Mangal et al., 2022; Munyaneza and Bizimungu, 2022). These pathogenic organisms are either soil or air-borne and usually inflict harm on all plant parts. The most commonly encountered diseases that affect potato growth and development include common scab, dry rot, black scurf, late blight, stem canker, premature deaths, and a wide array of nematodes. Soil-based infections that affect the quality of tubers include pink rot, common scab, leak, black scurf, powdery scab, black dot, Fusarium dry rot, and root knot nematode (Fiers et al., 2012; Hussain et al., 2021; Kowalska, 2021; Póss et al., 2021; Tegg and Wilson, 2022). However, it should be noted that *Rhizoctonia solani, Alterneria solani, Phytophthora infestans, Verticillium wilt*, and *Streptomyces scabies* rank highly among the most predominant re-emerging pathogens within the potato sector on a global scale (Muhammad et al., 2013; Abdurahman et al., 2019). Screening and detection of the aforementioned pathogens is deemed important for the planning of timely mitigation measures against these diseases, as well as predicting future outbreaks, especially within the asymptomatic zones (Hussain et al., 2017). Therefore, it is essential to develop effective, sensitive and credible diagnostic techniques that can detect resistant strains prior to the early onset of diseases (Islam et al., 2017; Kumar et al., 2019). Detection methods can utilize pathogen molecular markers, which additionally allows the distinction of strains within a given species, allowing farmers to adopt and embrace the most robust mitigation strategies before the emergence of disease symptoms. What's more, these same markers can be utilized for pre-plant seed health testing in order to guarantee that seed movement occurring across borders is only for the disease-free materials, which also limits the need for quarantine periods.

DNA-based technologies exemplified by techniques such as polymerase chain reaction (PCR), loop-mediated isothermal amplification (LAMP), quantitative-PCR (qPCR), and sequencing methods are often largely utilized in the identification and screening of plant-based pathogens (Bock et al., 2010; Hussain et al., 2014; Khan et al., 2018; Lees et al., 2019). What's more interesting, bioinformatics has made it possible for researchers in the field of plant pathology to identify sequences of DNA and their motifs, thereby elevating the precision of modern diagnostic techniques (Bock et al., 2010). Proteomics holds the potential of providing information regarding virulence factors and pathogenicity, which can subsequently lead to novel techniques in the diagnosis and screening of plant related diseases, while also simultaneously determining the most suitable protective measures. Cumulatively, practices that are centered around disease management, the invention of new disease-resistant cultivars and rapid, cost-effective pathogen diagnostic techniques are the fundamental approaches in the deterrence of devastating crop losses caused by these potato pathogens.

Potato ranks fourth as the world's widely consumed crop with its production totaling to 368 million tons annually. Despite the instrumental role played by the potato crop in the food chain, very scarce studies are available detailing the distribution and composition of endophytes in potato and their correlation with the associated diseases. Considered the largest soil-borne disease in the potato market, Potato Common Scab (PCS) is solely responsible for severe economic losses globally. PCS is known to cause both shallow and deep blemishes on potato surfaces (Leiminger et al., 2013; Arslan et al., 2018; Sarwar et al., 2018), which then negatively impact on the taste, palatability and quality of potatoes (Getahun, 2018). Given that PCS can be regulated using certain micro-organisms, the present study sought to investigate the distribution, composition, and occurrence of endophytes belonging to bacteria within potato tubers, roots and stems. The immediate response of these endophytes to PCS was also studied by conducting field trials in Jiaozhou City, Shandong Province, China. Sampling was done per plant with the stem, tuber, and roots collected for analysis. Analysis was done *via* high-throughput sequencing, and the composition of endophytes in all the aforementioned three plant parts showed significant differences (*p* < 0.05).

Distribution of bacteria-based communities additionally showed a gradual sloping from soil to root to tuber/stem (GS/RS to RE to TE/SE). The study was able to demonstrate that roots function as the gate pass for soil bacteria, a factor that explains 50% presence of root endophytes in potato soil communities. Additionally, the study illustrated that PCS significantly lessened the ACE and OBS indexes of root endophytes, without considerably diminishing the indexes of TE and SE. The specific PCS pathogen investigated in this research, OTU62, was found to be present in roots, stems, and tubers, even if the PCS symptoms were mild and strong networks of endophytes were present. Given these findings, the current study offers novel insights into the characteristic composition and distribution of endophytic communities in the potato crop and their immediate response to potato common scab.

Viral diseases are equally responsible for causing severe constraints on production systems of potatoes (Devaux et al., 2020; Kreuze et al., 2020), resulting in ~50% tuber yield reduction (Wale et al., 2008). Vegetative propagation, which is mainly utilized in potato reproducibility, often results in viral transmission over successive generations making the potato crop susceptible to numerous viral infections (Kreuze et al., 2020; Jones, bibx2014). At least 40 potato viruses have been documented globally, with the potato viruses X (PVX), S (PVS), M (PVM), Y (PVY), A (PVA), and potato leaf roll virus (PLRV) being the principal pathogens responsible for majority of yield reductions in the potato crop (Zheng et al., 2010; Awasthi and Verma, 2017; Kreuze et al., 2020). Given the remarkable advancements made in the identification of plant viruses, it has become possible to utilize novel molecular diagnostic tools to accelerate virus detection and screening (Maclot et al., 2020). In this study, potato viromes were identified using total RNA-sequencing, with 22 libraries being prepared from leaves, stems, roots, tubers and stolons. The latter parts were sampled from 5 cultivars bin native landraces of Liangshan and some from Russia, all of which were virus-free at the time of collection.

The study aimed at addressing the ramifications of indigenous viruses on both native and newly-acquired potato germplasms by comparing viromes belonging to diverse varieties and later characterizing the host-specific viral communities in a tissue-level atlas. Data analysis of viral genome sequences was done using bioinformatics through which the complexity and uniqueness of viral communities was revealed. The results of this study showed that potato viruses PVS, PVY, and PVM were the most dominant and frequently encountered viruses in the field, with infection bias being observed in both indigenous and infected cultivars. Tissue-specific infection was also observed in which underground parts of the potato plant (tubers ad roots) harbored more unusual viruses. Sequence variation in viruses showed higher frequency of the single nucleotide polymorphism more than other tissues. The findings of the present study establish firm foundations of viral disease control strategies in potato farming for future cultivation and production.

The potato industry ranks among one of the leading food sectors that is heavily relied upon for nourishment and sustenance of the global populace (Soare and Chiurciu, 2021). Despite its importance, the potato plant remains predisposed to pathogen and pest invasion, including severe viruses and viroids (Harahagazwe et al., 2018). Amongst these viruses, PVY (Potyviridae), which contains approximately 160 species (ICTV Report on Virus Classification Taxon Nomenclature, 2020, ranks in the fifth position in terms of the global top ten important plant viruses (Scholthof et al., 2011) considered to be the most economically potent (Valkonen, bibx2007). Infections caused by PVY on potatoes manifest as either mild to severe symptoms including yellows, mosaic, mottling, rogues, necrosis, leaf malformation, and plant defoliation (Valkonen, bibx2007). The intensity and type of the aforementioned symptoms varies widely based on prevailing environmental conditions and genetic diversity of the PVY strain (Lacomme and Jacquot, 2017). Severe economic impact of the PVY on potato yield has been reported in various countries including Ireland (16.5%), Canada (34%), India (30–40%), Kenya (37%), America and Poland (40–44%), and also China (50%) (Gray et al., 2010; Were et al., 2013; Hutton et al., 2015; Jailani et al., 2017).

Regardless of numerous mitigation strategies being implemented for PVY eradication, it remains incurable under field conditions. The most promising approach in dealing with this viral infection is the molecular approach, in which understanding the molecular response and molecular aspects of PVY infection, genome organization, and PVY enconded proteins is paramount. Bioinformatics tools have shown promising results in monitoring viral transmission and demonstrating the impact of PVY on infected potato cultivars. Given that PVY transmission dynamics are highly complex, more intensive work needs to be done in order to better understand its mechanisms at a molecular, biochemical, and bioinformatics level. The present review sought to present current knowledge on PVY viral transmission from a molecular to bioinformatics approach, focusing on the epidemiology, genome organization and effective management of this viral disease in the potato plant.

Early blight (EB) is a lethal plant disease caused by the filamentous fungus *Alternaria solani* and causes devastating yield losses in potato crops. Most often, EB infects plant parts above ground, with symptoms manifesting as either small brown lesions or large ones that grow on leaves (Dhaval et al., 2021). Under favorable climate, these lesions normally enlarge to form concentric-like rings often engulfed by yellowish like halos (Bessadat et al., 2017; Dhaval et al., 2021). Conidia is the primary inoculum of *A. solani* and therefore, the pathogen is aerially transmitted with infections spreading from infected to uninfected leaves *via* wind, insects, or splash (Pandey et al., 2021). Upon maturity, these conidiospores form germ tubes, which then invade the host plant tissues occasionally (Mamgain et al., 2013; Jindo et al., 2021; Mushrif et al., 2021) The distinctive feature of *A. solani* conidium being airborne renders it problematic to control and manage its occurrence in potato plants. Therefore, accurate and timely detection of this fungal pathogen at the infection stage is very crucial for the forecasting of epidemics associate with EB. The current study sought to develop an RNA-based approach, which is both highly sensitive and precise in the detection of *A. solani* in whole potato leaves at a single spore level, based on quantitative real-time PCR. Twenty-two fungal isolates of *A. solani* were sourced from Hebei Agricultural University, alongside other supplementary fungal and bacterial species sourced from China. Primers specific to *A. solani* were designed followed by RNA extraction, DNA digestion and quantitative real-time PCR. The study was able to develop an RNA-based method for the specific and sensitive detection of this fungal pathogen. The main breakthrough of this research was the discovery of a new gene, jg1677, which is specifically and stably expressed by *A. solani*. Using the 22 sourced pathogens including *A. solani*, the study was able to verify that jg1677 is specific for *A. solani* and therefore, can be used to detect EB disease. The invented method can be used to detect as minute as one spore infesting a potato leaf or leaves. Conclusively, the adoption of this innovative technology can enormously assist in the screening, identification and detection of pathogens whilst introducing novel curbing measures for disease epidemics; thereby leading to increased food availability.

## Author contributions

All authors listed have made a substantial, direct, and intellectual contribution to the work and approved it for publication.
